# Elderly abuse and anxiety symptoms among older adults in China: the mechanism of physical frailty and social frailty

**DOI:** 10.3389/fpsyt.2026.1813570

**Published:** 2026-10-08

**Authors:** Wenle Zhang, Wen Hou, Zhu Guo, Jiaxin Feng, Baoyi Liao, Jiahong Xie, Dongli Wang, Rui Gao, Maomin Jiang, Yisong Yao

**Affiliations:** 1 Xiamen Xian-yue Hospital, Xian-yue Hospital Affiliated with Xiamen Medical College, Fujian Psychiatric Center, Fujian Clinical Research Center for Mental Disorders, Xiamen, China; 2 Department of Sports and Health Sciences, Faculty of Arts and Social Sciences, Hong Kong Baptist University, Hong Kong, Hong Kong SAR, China; 3 School of Nursing, Suzhou Medical College of Soochow University, Suzhou, China; 4 Key Laboratory of Digital-Intelligent Disease Surveillance and Health Governance, North Sichuan Medical College, Nanchong, China; 5 The Primary Health Development Research Center of Sichuan Province, Nanchong, China

**Keywords:** anxiety symptoms, elderly abuse, older adult, physical frailty, social frailty

## Abstract

**Purpose:**

This study aims to examine the association between elderly abuse and anxiety symptoms among older adults and explore the mechanisms of physical frailty and social frailty between them.

**Methods:**

A total of 1361 older adults were included in this study. Multiple linear regression and propensity score matching was conducted to confirm the association between elderly abuse and anxiety symptoms. Mediation and moderation analyses were performed to explore the mechanisms of physical frailty and social frailty between elderly abuse and anxiety symptoms.

**Results:**

Elderly abuse was significantly associated with higher levels of anxiety symptoms. Psychological abuse and neglect showed the strongest associations with anxiety symptoms. Social frailty played moderating roles between both psychological abuse, neglect and anxiety symptoms, while physical frailty played moderating roles between only psychological abuse and anxiety symptoms. Physical frailty and social frailty both partly mediated the relationship between psychological abuse, neglect and anxiety symptoms.

**Conclusions:**

This study provides insight into the systematic relationship between elderly abuse and anxiety symptoms among older adults in Sichuan province. Preventive strategies should address abuse, physical health, and social support simultaneously of older adults.

## 1 Introduction

Population aging has been a significant trend in the 21st century, and the global aging trend affects developing countries more than developed countries (1). The 2020 national census revealed that the population aged 60 and older surpassed 260 million, accounting for 18.7% of the total population in China (2). Projections indicate that by 2050, the population of older adults in China will exceed 450 million (3). Older adults are especially vulnerable to mental health disabilities (4). Therefore, attention to this stage of life, particularly emotional well-being, is necessary.

Anxiety symptoms and disorders are common in older adults and often go undetected (5). A recent study by Zeng et al. showed that the age-standardized disability-adjusted life years rate for anxiety disorders in middle-aged and older adults in China has shown adverse trends (6). Previous studies have shown that biological processes of aging may contribute to poor mental health and are a potential risk factor for anxiety in middle-aged and older adults (7). Moreover, serious public health problems, such as social isolation among older adults, also increase their risk of mental health problems (8). Therefore, in addition to focusing on high-risk groups, greater attention should be paid to elucidating the reasons for anxiety symptoms in older adults.

In addition, elderly abuse, a serious threat to public health, is a growing global trend that increases risk factors for older adults’ health (9). However, elderly abuse remains a neglected issue in both health policy and research and is underreported, meaning the problem may be even more severe than anticipated (10). Elderly abuse is regarded as a single or repeated act, or the lack of appropriate action, occurring within any relationship where there is an expectation of trust, and such an act or lack of action causes harm or distress to an older adult (9). According to a 2021 World Health Organization (WHO) report, one in six older adults experiences abuse (11). A cross-sectional study based on a sample of 7,466 adults randomly recruited from six regions in China demonstrated that one in 10 participants reported that their elderly parents had experienced abuse or neglect in the past year (12). The elder victims were reported to have suffered abuse or neglect on multiple occasions, with the frequency of abuse incidents ranging from 3.6 to 11.82 occasions per participant in the past year (12). A small-scale study also reported prevalence rates of 11.48% and 8.24% in Macau and Guangzhou, respectively (10). The pandemic of elder abuse has made the topic a critical research area, especially in the fields of gerontology and public health.

Many studies have shown that older adults who have experienced any form of abuse have poorer mental health and are more likely to develop mental disorders, including anxiety, than those who have not experienced abuse (13, 14). Gursoy et al. reported that exposure to psychological abuse, economic abuse, and neglect had statistically significant effects on generalized anxiety disorders among older adults in Turkey (15). Results from a national longitudinal study also showed that mistreated older adults had more anxiety symptoms (16). Furthermore, exposure to elder abuse was linked to specific types of anxiety among older adults, such as death anxiety and SARS-CoV-2 Omicron-induced anxiety (17, 18). However, no study to date has examined the relationship between elderly abuse and generalized anxiety symptoms in the context of the Chinese older adult population. Although the broad association between elder abuse and anxiety is already well established, a few questions remain open. First, most existing evidence comes from Western samples, and it is unclear whether the abuse–anxiety link holds similarly among Chinese community-dwelling older adults (19–21). Second, few studies have examined whether different abuse subtypes (psychological, physical, financial, neglect) show differential associations with anxiety (15, 20, 22). Third, the role of frailty-already common in this population-as a potential mediator or moderator of this relationship has received limited direct testing (23–25).

Accordingly, the first hypothesis is: a significant positive relationship exists between elder abuse and elevated anxiety levels among older adults in China, with psychological abuse and neglect expected to show stronger associations with anxiety compared to physical abuse and financial exploitation.

Frailty is regarded as a multidimensional biopsychosocial syndrome, usually divided into physical and social frailty (26). Physical frailty is an age-related state of reduced physiological reserve (27). Social frailty—which can be defined across several domains—describes a deficiency in the quality or quantity of social connections, or the degree of support, available to individuals (28). Physical and social frailty are global phenomena, with high prevalence and consistent associations across different countries. The high prevalence rates, along with evidence of worse clinical outcomes when physical and social frailty coincide, emphasize the importance of measures to address these phenomena (29).

Several studies have reported a particularly strong association between elder abuse and physical frailty in older adults (30, 31). A study from Mexico further found that frailty was associated with conflict-related abuse, including physical, psychological, and sexual abuse (23). In addition, older adults with dense social networks had a lower risk of elder abuse (32). Yang et al. (33) also evaluated the relationship between different types of social participation and elder abuse. Formal social participation was associated with psychological abuse, while weekly informal socializing was associated with psychological abuse and financial exploitation among women. Beyond these established abuse–frailty links, frailty is consistently associated with anxiety. A systematic review has demonstrated a clear association between physical frailty and anxiety in older adults (34). The importance of social connectedness and, conversely, social frailty, in the etiology of anxiety symptoms has been documented extensively in the previous literature and in theories of perceived isolation (35). Notably, China harbors the world’s largest older adult population, with rapidly rising frailty prevalence, yet empirical evidence on the frailty–anxiety link within this specific context remains limited.

Although the frailty–anxiety association is well documented (34, 35), the literature offers limited guidance on the specific role frailty plays within the abuse–anxiety link. Two non-exclusive possibilities are worth distinguishing. First, frailty may operate as a mediator: abusive experiences can erode an older adult’s physical and social capacities, and the resulting frailty, in turn, increases susceptibility to anxiety. Second, frailty may operate as a moderator: older adults who are already frail possess fewer physiological and social reserves to absorb the impact of abuse, such that the same exposure produces a stronger anxiety response.

Therefore, the second hypothesis is: physical frailty and social frailty each act as both a mediator and a moderator in the relationship between elderly abuse and anxiety symptoms among older adults.

This study aims to examine the association between elder abuse and anxiety symptoms among older adults in China and further explore the potential mechanisms through which physical and social frailty operate. The findings of this study can provide a robust theoretical foundation for the development of intervention measures, thereby promoting the mental health of older adults.

## 2 Methods

### 2.1 Study design and participation

This study was based on a cohort named Whole Life Cycle Health Investigation of Sichuan Province Older Adults (LCIOA-2025). The random number table method was used to choose eight different districts (counties) in each cohort. Quota sampling was conducted according to gender and regional population size. Including criteria: (1) ≥60 years; (2) Be able to complete the survey; (3) Be resident in Sichuan Province; (4) Give informed consent and participate voluntarily in the study. LCIOA cohort was conducted in accordance with the Declaration of Helsinki and approved by the Ethics Committee of North Sichuan Medical College and the Ethics Committee of Xiamen Xian-yue Hospital. In this study, a total of 1361 older adults were included.

### 2.2 Measures

#### 2.2.1 Elderly abuse

Elderly abuse was assessed using seven items adapted from a previously validated measure in China (14). Respondents were asked whether they had experienced any abusive behaviors from family members over the past year, encompassing four dimensions: psychological abuse, physical abuse, neglect and financial exploitation. Each item was coded dichotomously (0 = No, 1 = Yes), and scores were summed to yield a total abuse score ranging from 0 to 7, with higher scores indicating greater levels of abuse. The internal consistency of the scale was acceptable, with a Cronbach’s alpha of 0.773.

#### 2.2.2 The generalized anxiety disorder-7

Anxiety symptoms were assessed through GAD-7, which is a self-report anxiety symptoms scale to describe the feelings of the past two weeks (36). There are 7-item in the scale and assess anxiety by calculating the total score. Each item is scored from 0 to 3, and the total score ranged from 0 to 21. A higher score indicates a higher level of anxiety symptoms. The Cronbach’s α of the GAD-7 was 0.819 in this study.

#### 2.2.3 The fatigue, resistance, ambulation, illnesses, and loss of weight scale

Physical frailty was evaluated using the FRAIL scale, which has been validated in Chinese older adult populations (37, 38). Fatigue and unexplained weight loss were identified if participants answered “yes” to the questions: “Were you tired most of last week?” and “Have you lost more than 5% of your body weight in the last year without any clear reason?” Resistance and ambulation deficits were identified by negative responses to “Can you climb a staircase?” and “Can you walk 500 meters?” respectively. Illness was measured by the presence of at least five chronic diseases, which was then converted into a binary variable. Each of these five variables was coded as 0 (“no”) or 1 (“yes”), with 1 indicating a deficit. The total number of deficits was aggregated to compute a frailty score ranging from 0 to 5. A higher score indicates a higher level of physical frailty. The Cronbach’s α of the FRAIL was 0.744 in this study.

#### 2.2.4 The lubben social network scale

Social frailty was measured by LSNS, which has been cross-validated in a Chinese context (39, 40). The LSNS included three questions assessing family relationships and a similar three questions on friendships. Items were rated on a 0–5 Likert scale, with options ranging from “none” to “nine or more”. Total scores ranged from zero to 15 points. A higher score indicates a lower level of social frailty. The Cronbach’s α for family isolation was 0.747, and for friend isolation was 0.827.

#### 2.2.5 Covariate variables

We included age, sex, urban-rural distribution, income per month, education level, living alone, marital status, chronic diseases.

### 2.3 Statistical analysis

In this study, we employed t-tests and analysis of variance (ANOVA) for descriptive statistical analysis. Subsequently, we utilized correlation analysis to assess the potential correlations between anxiety symptoms and other variables. We then explored the impact of elderly abuse and its four dimensions on anxiety symptoms using multiple linear regression analyses. Propensity score matching (PSM) was performed in R 4.6.0 using the MatchIt package; propensity scores were estimated by logistic regression on 15 covariates, and three matching approaches were used: 1:1 nearest-neighbor matching, 1:4 nearest-neighbor matching, and radius matching (caliper = 0.02, without replacement). Covariate balance was assessed using standardized mean differences (SMD < 0.10). Then, we used the PROCESS macro 4.1 for SPSS (Model 4) to analyze the mediating effects of physical frailty and social frailty on the significant association between dimensions of elderly abuse and anxiety symptoms, employing the bootstrap method with 5,000 resamples and 95% bias-corrected confidence intervals to validate the mediation effect. We also used PROCESS Model 1 to explore whether physical frailty and social frailty have moderating effects on the significant association between dimensions of elderly abuse and anxiety symptoms. As a sensitivity analysis, the Bonferroni correction was applied within the mediation analyses (10 tests; α = 0.005), the moderation analyses (10 tests; α = 0.005), and the subgroup analyses (4 tests; α = 0.0125). Data were analyzed using SPSS 26.0 and PROCESS 4.1, with a *P* value < 0.05 considered statistically significant.

## 3 Results

### 3.1 Common method bias test

Common method bias test was applied to all scale items, resulting in the extraction of six factors with eigenvalues greater than 1. The cumulative variance explained by the first factor was 27.838%, which is below the critical threshold of 40%. Thus, no significant common method bias was found in this study.

### 3.2 Descriptive statistics

A total of 1361 older adults were included in this study. Among them, 641 of them (47.1%) were males, and 663 of them (48.7%) were from urban. The mean score of anxiety differed from different urban-rural distribution, age, income per month, education level, marital status and chronic diseases group (*P* < 0.05). More details were shown in **Table 1**.

**Table 1:** Descriptive statistics.

Variables	Characteristics			
	N (%)	M ± SD	t/H	P
Sex			-1.410	0.159
Male	641 (47.1%)	11.29 ± 4.410		
Female	720 (52.9%)	11.63 ± 4.449		
Urban-rural distribution			-8.093	<0.001
Urban	663 (48.7%)	10.49 ± 4.051		
Rural	698 (51.3%)	12.39 ± 4.581		
Age			8.998	<0.001
60∼69 years	730 (53.6%)	11.26 ± 4.528		
70∼79 years	465 (34.2%)	11.30 ± 4.177		
≥80 years	166 (12.2%)	12.83 ± 4.486		
Income per month			7.808	<0.001
3000 and below	867 (63.7%)	11.66 ± 4.289		
3001∼5000	305 (22.4%)	10.98 ± 4.39		
5001∼10000	140 (10.3%)	11.29 ± 5.157		
10001 and up	49 (3.6%)	11.65 ± 4.824		
Education level			1.626	0.197
Junior high school and below	984 (72.3%)	11.44 ± 4.289		
Senior high school/technical secondary school/technical school	241 (17.7%)	11.83 ± 4.711		
College degree and above	136 (10.0%)	10.99 ± 4.899		
Living alone			0.281	0.779
Yes	165 (12.1%)	11.56 ± 4.448		
No	1196 (87.9%)	11.45 ± 4.432		
Marital status			-4.215	<0.001
Yes	997 (73.3%)	11.16 ± 4.339		
No	364 (26.7%)	12.30 ± 4.581		
Chronic diseases			3.117	0.002
No	1138 (83.6%)	11.63 ± 4.444		
Yes	223 (16.4%)	10.62 ± 4.282		

M ± SD, mean ± standard deviation; t, statistic of the independent-samples t-test; H, statistic of the Kruskal–Wallis test; P, P value.

### 3.3 Correlation analysis

The results of Pearson correlation analysis indicated that social frailty was negatively correlated with physical frailty (r=-0.197, *P* < 0.01); negatively correlated with anxiety (r=-0.106, *P* < 0.01); and negatively correlated with elderly abuse (r=-0.113, *P* < 0.01). Physical frailty showed a strong positive correlation with anxiety (r=0.689, *P* < 0.01) and a positive correlation with elderly abuse (r=0.285, *P* < 0.001). Anxiety was positively correlated with elderly abuse (r=0.282, *P* < 0.01).

Among the specific types of elderly abuse, psychological abuse, financial exploitation, neglect, and physical abuse were all negatively correlated with social frailty and positively correlated with both physical frailty and anxiety, with statistically significant results (*P* < 0.01). More details were shown in **Supplementary Table 1**.

### 3.4 Relationship between elderly abuse and anxiety among older adults

To evaluate the relationship between elderly abuse and anxiety among older adults, we built two linear regression models. Model 1 included all five abuse dimensions and the covariates; Model 2 included the two dimensions that were significantly associated with anxiety in Model 1 (psychological abuse and neglect) and the covariates. Model 2 showed the significant association between psychological abuse (B = 0.797, t=2.245), neglect (B = 1.298, t=2.090) and anxiety among older adults (P<0.05). More details were shown in **Table 2**.

**Table 2:** Linear regression of elderly abuse and anxiety among older adults.

Variables	Model 1		Model 2	
	B	t	B	t
Constant	5.560	2.282^*^	6.311	2.513^*^
Urban-rural distribution	1.675	6.631^**^	1.970	7.607^**^
Age	0.295	1.735	0.353	2.020^*^
Income per month	0.278	1.557	0.237	1.288
Education level	-0.038	-0.326	0.024	0.007
Marital status	0.797	2.95^*^	0.950	3.415^*^
Elderly abuse	0.971	1.250	–	–
Psychological abuse	0.780	2.188^*^	–	–
Financial exploitation	0.171	0.278	–	–
Neglect	1.211	1.944^*^	–	–
Physical abuse	-0.886	-1.300	–	–
*R*	0.364		0.265	
*R²*	0.133		0.070	
*F*	20.719**		20.258**	

*P < 0.05, **P < 0.001. B, unstandardized regression coefficient; t, t-statistic; R, multiple correlation coefficient; R², coefficient of determination; F, F-statistic.

Although the associations were statistically significant, the regression models explained only a limited proportion of the variance in anxiety symptoms (R² = 0.133 and 0.070 for Models 1 and 2, respectively), suggesting that anxiety symptoms are influenced by multiple factors. These findings should therefore be interpreted with caution.

To confirm the robustness of the relationship between elderly abuse and anxiety, we performed three PSM methods. The 95% CIs for the Average Treatment Effects on Treated (ATT) under all matching methods did not include zero, and the *P* values were less than 0.05, indicating that there was statistically association between elderly abuse and anxiety symptoms. After controlling for other covariates, individuals who experienced abuse had an average anxiety symptom score approximately 1.11 to 1.65 points higher than similar individuals who did not experience abuse. More details were shown in **Supplementary Table 2**.

### 3.5 Elderly abuse and anxiety symptoms among different characteristics of older adults

There was significant association between psychological abuse and anxiety symptoms among older males (β=1.653, P = 0.001; **Supplementary Table 3**). Among older females, the association between elderly abuse and anxiety symptoms was significant ((β=2.379, *P* < 0.05; **Supplementary Table 3**). There was significant association between psychological abuse and anxiety symptoms among older adults who lived in urban (β=1.171, *P* < 0.01; **Supplementary Table 4**). Among older adults who lived in rural, the association between elderly abuse and anxiety symptoms was significant (β=3.418, *P* < 0.01; **Supplementary Table 4**).

### 3.6 The mechanism between elderly abuse and anxiety symptoms among older adults

#### 3.6.1 The moderating effect of physical frailty and social frailty

The results showed that the moderating effect of physical frailty and social frailty had significant effects on the association between psychological abuse and anxiety symptoms (Coef=0.042, *P* < 0.01; Coef=0.153, *P* < 0.001; **Supplementary Table 5**; **Figure 1A**). When the Bonferroni correction (α = 0.005) was applied as a sensitivity analysis, the moderating effect of social frailty on the psychological abuse–anxiety association remained significant, whereas the moderating effect of physical frailty (P = 0.007) was attenuated to non-significance. The results showed that the moderating effect of social frailty had significant effects on the association between neglect and anxiety symptoms (Coef=0.269, *P* < 0.001; **Supplementary Table 6**; **Figure 1B**).

**Figure 1:**
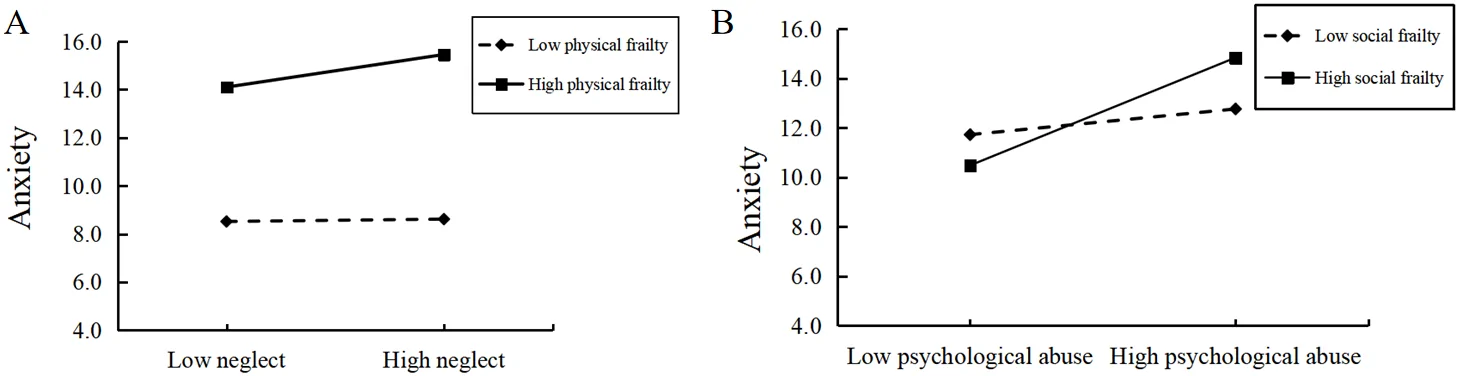
**(A)** The moderating effect between psychological abuse and anxiety symptoms; **(B)** The moderating effect between neglect and anxiety symptoms. In panel **(A)**, dashed and solid lines represent low (-1 SD) and high (+1 SD) physical frailty, respectively; in panel **(B)**, dashed and solid lines represent low (-1 SD) and high (+1 SD) social frailty, respectively.

#### 3.6.2 The mediating effect of physical frailty and social frailty

We used Bootstrap method to test the accuracy of the results and showed that the direct effect of psychological abuse on anxiety symptoms was 0.397 (95% CI=[0.153, 0.640]) and the indirect effect of physical frailty on psychological abuse and anxiety symptoms was 0.820 (95% CI=[0.588, 1.047]). The total effect of psychological abuse on anxiety symptoms was 1.217 (95% CI=[0.911, 1.523]), with 32.62% direct and 67.38% indirect. The confidence intervals for the total, indirect, and direct effects did not include 0, which further suggests that psychological abuse was associated with higher anxiety symptoms, partly explained by physical frailty. See **Table 3** for details.

**Table 3:** The mediatingse, neglect and anxiety symptoms.

Variables	Pathway	Effect	SE	Proportion (%)	95% CI	
					Lower	Upper
Direct effect a	Psychological abuse→Anxiety symptoms	0.397	0.124	32.62	0.153	0.640
Indirect effect a	Psychological abuse→Physical frailty→Anxiety symptoms	0.820	0.116	67.38	0.588	1.047
Direct effect b	Psychological abuse→Anxiety symptoms	1.178	0.156	96.80	0.872	1.484
Indirect effect b	Psychological abuse→Social frailty→Anxiety symptoms	0.039	0.019	3.20	0.008	0.081
Total effect	–	1.217	0.156	100	0.911	1.523
Direct effect c	Neglect→Anxiety symptoms	0.982	0.253	41.24	0.485	1.479
Indirect effect c	Neglect→Physical frailty→Anxiety symptoms	1.399	0.234	58.76	0.937	1.881
Direct effect d	Neglect→Anxiety symptoms	2.287	0.323	96.05	1.654	2.920
Indirect effect d	Neglect→Social frailty→Anxiety symptoms	0.094	0.041	3.95	0.021	0.185
Total effect	–	2.381	0.322	100	1.749	3.012

Direct and indirect effects are unstandardized regression coefficients (B), representing mean changes in the GAD-7 total score; SE, standard error; 95% CI, 95% bias-corrected and accelerated bootstrap confidence interval.

For social frailty, the direct effect was 1.178 (95% CI = [0.872, 1.484]), and the indirect effect was 0.039 (95% CI = [0.008, 0.081]). The total effect of psychological abuse on anxiety symptoms was 1.217 (95% CI=[0.911, 1.523]), with 96.80% direct and 3.20% indirect. The confidence intervals for the total, indirect, and direct effects did not include 0, which further suggests that psychological abuse was associated with higher anxiety symptoms, partly explained by social frailty. See **Table 3** for details.

The results showed that the direct effect of neglect on anxiety symptoms was 0.982 (95% CI=[0.485, 1.479]) and the indirect effect of physical frailty on neglect and anxiety symptoms was 1.399 (95% CI=[0.937 1.881]). The total effect of neglect on anxiety symptoms was 2.381 (95% CI=[1.749, 3.012]), with 41.24% direct and 58.76% indirect. The confidence intervals for the total, indirect, and direct effects did not include 0, which further suggests that neglect was associated with higher anxiety symptoms, partly explained by physical frailty. See **Table 3** for details.

For social frailty, the direct effect was 2.287 (95% CI=[1.654, 2.920]), and the indirect effect was 0.094 (95% CI = [0.021 0. 185]). The total effect of neglect on anxiety symptoms was 2.381 (95% CI=[1.749, 3.012]), with 96.05% direct and 3.95% indirect. The confidence intervals for the total, indirect, and direct effects did not include 0, which further suggests that neglect was associated with higher anxiety symptoms, partly explained by social frailty. See **Table 3** for details. All effects are unstandardized regression coefficients representing mean changes in the GAD-7 total score.

## 4 Discussion

This study revealed a significant association between elder abuse and anxiety symptoms among older adults. Among different forms of abuse, psychological abuse and neglect showed the strongest associations with anxiety symptoms. Social frailty played a moderating role between psychological abuse, neglect, and anxiety symptoms, while physical frailty played a moderating role only between psychological abuse and anxiety symptoms. Physical frailty and social frailty both partially mediated the relationship between psychological abuse, neglect, and anxiety symptoms. These findings help clarify the patterns of association and potential pathways linking elder abuse, physical frailty, social frailty, and anxiety symptoms among older adults.

This study found a significant association between elder abuse and anxiety symptoms among older adults, and this association remained robust across multiple PSM strategies. This finding aligns with prior evidence indicating that exposure to abuse is linked to a range of adverse mental health outcomes in later life, especially anxiety among community-dwelling older adults (15, 41–43). Across cultural contexts, older adults who report abuse consistently exhibit poorer emotional well-being than those without such exposure (44–46). Further analyses in the present study indicated that the strength of the association with anxiety symptoms varied by abuse subtype, with psychological abuse and neglect showing stronger associations than other forms after adjustment for relevant covariates. Similar subtype-specific patterns have been reported in community-based studies across diverse cultural settings, in which non-physical forms of abuse show more consistent associations with anxiety and emotional distress after adjustment for sociodemographic factors (47, 48). Overall, elder abuse is associated with anxiety symptoms in later life and shows a patterned relationship across abuse subtypes, pointing to the central role of individual vulnerability in shaping anxiety responses to abuse exposure.

Physical frailty emerged as a major mediator of the associations between psychological abuse or neglect and anxiety symptoms among older adults. In the present study, physical frailty emerged as a major mediator of the associations between psychological abuse or neglect and anxiety symptoms among older adults. In the present study, physical frailty explained a notable proportion of the observed association between abuse exposure and anxiety symptoms in this cross-sectional sample, and physical frailty itself showed a strong independent association with anxiety. This pattern is consistent with the interpretation that anxiety symptoms in the context of elder abuse may be closely intertwined with underlying physical vulnerability, although the cross-sectional design precludes causal inference, and bidirectional relationships remain plausible.

Physical frailty has consistently been linked to both elder abuse and adverse emotional outcomes in later life, supporting its relevance as a vulnerability-related factor connecting these experiences (24, 34, 49). Physical frailty reflects diminished physiological reserve and heightened functional vulnerability in later life (50, 51).

Functional impairment and dependence have been linked to greater severity and broader exposure to elder abuse and neglect, while psychosocial distress is common among frail older adults (52, 53). Within this framework, physical frailty may contribute to the translation of adverse interpersonal experiences into heightened anxiety by amplifying concerns about health-related uncertainty and perceived threats to functional independence, which are common among frail older adults (54). In the present study, physical frailty was assessed using indicators of fatigue, impaired mobility, resistance limitations, illness burden, and unintentional weight loss. Collectively, these features reflect diminished physical resilience and functional vulnerability (37). Such a state may heighten concerns about bodily integrity, daily functioning, and future dependence, thereby increasing vulnerability to persistent worry and heightened arousal when older adults are exposed to psychological abuse or neglect (16). Rather than constituting a discrete psychological mechanism, physical frailty is better conceptualized as a broader condition in which physiological vulnerability and emotional distress may reinforce one another.

Differential findings across abuse subtypes. Physical abuse and financial exploitation showed no significant association with anxiety after covariate adjustment. This null finding may reflect several possibilities. First, the relatively low prevalence of these abuse types in our sample limited statistical power to detect associations. Second, physical abuse may manifest through other mental health outcomes, such as post-traumatic stress symptoms, pain-related distress, or fear-based responses, rather than generalized anxiety as measured by GAD-7 (55–57). Third, financial exploitation’s psychological impact may operate through delayed or alternative emotional pathways (e.g., depression, anger, or feelings of betrayal) not captured by the anxiety measure used here (58–62). Additionally, the moderation patterns differed across frailty types: physical frailty moderated only the effect of psychological abuse on anxiety, whereas social frailty moderated both psychological abuse and neglect. This asymmetry may reflect that physically frail older adults, who often retain cognitive awareness of their declining capacity, are particularly vulnerable to the psychological distress induced by verbal or emotional abuse (20, 23, 63–65). In contrast, socially frail individuals lack the buffering effect of social support, rendering them susceptible to both psychological abuse and the consequences of neglect. These subtype-specific patterns suggest that abuse subtypes operate through partially distinct mechanisms, warranting further investigation in larger, abuse-type-stratified samples.

It should be noted that the strong association observed between physical frailty and anxiety symptoms may partly reflect overlap between the somatic components of frailty and the physical manifestations of anxiety, such as fatigue and reduced activity (34, 66). This overlap has implications for interpreting the mediation findings: the somatic similarity may inflate the bivariate correlation between physical frailty and anxiety (r = 0.689) and, consequently, overestimate the apparent mediating role of physical frailty in the abuse–anxiety relationship. The mediation percentages reported (e.g., physical frailty accounting for a substantial share of the association) should therefore be interpreted with caution, as they reflect statistical decomposition in the presence of potential measurement overlap rather than a pure mechanistic pathway. Nevertheless, physical frailty and anxiety symptoms remain conceptually distinct constructs, representing physiological vulnerability and psychological distress, respectively. Moreover, anxiety symptoms may also contribute to subsequent declines in physical functioning, underscoring the potential for bidirectional relationships that warrant examination in longitudinal research (67, 68). Future studies could address this measurement issue by employing multidimensional anxiety measures that separate cognitive and somatic symptoms, or by using objective physical performance tests alongside frailty screening tools to reduce construct overlap.

Social frailty showed a limited mediating effect in the associations between psychological abuse or neglect and anxiety symptoms, accounting for only a small share of the observed relationship. In contrast to physical frailty, social frailty appears less likely to serve as a primary explanatory pathway linking abuse exposure to anxiety symptoms. More importantly, social frailty significantly moderated these associations, indicating that their strength varied across levels of social frailty. Social frailty primarily reflects the availability of social connections and interpersonal resources, such as the size of social networks and the frequency of contact with family members and friends (69, 70). Within the broader literature, social connectedness is commonly conceptualized as a contextual condition that influences how strongly stressful experiences affect emotional well-being, rather than as a factor that directly gives rise to psychological symptoms (71, 72). Older adults with higher levels of social frailty may have fewer opportunities to obtain emotional support or reassurance when exposed to adverse interpersonal experiences, thereby reducing their capacity to buffer psychological stress and increasing vulnerability to anxiety symptoms (73–75). In contrast to physical frailty, which reflects individual physiological vulnerability, social frailty operates at a relational and contextual level, shaping the intensity of anxiety responses to abuse or neglect rather than explaining the occurrence of anxiety itself. From this perspective, social frailty may shape the observed strength of the abuse–anxiety association in cross-sectional data. The moderating pattern observed here is consistent with a role of social resources in buffering or exacerbating emotional responses to abuse, although longitudinal data are needed to confirm whether social frailty functions as a true vulnerability factor or a correlate of other contextual influences.

The present study further examined whether associations between elder abuse and anxiety symptoms varied by demographic and contextual characteristics, including sex, age, and place of residence. These associations were broadly consistent across sex and age groups, with no clear differences observed. This consistency suggests that the relationship between elder abuse and anxiety symptoms is not confined to specific demographic subgroups. In contrast, differences were observed across residential settings. Older adults in different areas showed distinct patterns of association between psychological abuse or neglect and anxiety symptoms, suggesting that the strength of these associations may vary by place of residence. Place of residence may reflect broader social and environmental contexts, and older adults in different residential environments may differ in their access to social support, healthcare services, and community-based resources (76, 77). Such contextual differences may shape individuals’ capacity to cope with adverse interpersonal experiences, thereby contributing to variation in anxiety responses (71, 74, 78).

However, several limitations should be noted. First, the cross-sectional design precludes conclusions about causality or directionality among elderly abuse, physical frailty, social frailty, and anxiety symptoms. Although mediation and moderation analyses were conducted, the temporal order of these associations cannot be established. Longitudinal studies are needed to clarify causal pathways. Second, reliance on self-reported measures may have introduced self-report bias. Third, unmeasured confounders may still influence both experiences of elderly abuse and anxiety symptoms. Consistent with this, the regression models explained only modest variance in anxiety symptoms (R² = 0.057–0.121), indicating substantial contributions from unmeasured factors. Finally, physical frailty and social frailty were measured using screening or proxy indicators. In particular, social frailty reflects structural aspects of social networks rather than specific forms or quality of social engagement. Future studies should further examine how different dimensions of physical vulnerability and social relationships relate to anxiety outcomes among older adults.

The linear regression models explained only a modest proportion of the variance in anxiety symptoms (R² = 0.057–0.121), indicating that elder abuse and frailty account for only a limited part of the variation and that other unmeasured factors—such as chronic diseases, cognitive function, and personality traits—contribute substantially to anxiety in this population.

This study systematically evaluated associations and underlying mechanisms linking elder abuse, its various dimensions, and anxiety symptoms, with a particular focus on the mediating and moderating roles of physical and social frailty. The main findings highlight the significant relevance of psychological abuse and neglect to anxiety symptoms among older adults, with physical frailty identified as a central mediator in this relationship. In contrast, social frailty exhibited a limited mediating effect but demonstrated a significant moderating influence.

Although the cross-sectional design limits causal inference and the measurement indicators warrant further refinement, this research provides an integrative framework that incorporates physiological, psychological, and social factors to understand the mental health consequences of elder abuse in later life. Future studies should employ longitudinal designs to clarify the temporal sequence and bidirectional relationships among variables and further refine the examination of environmental characteristics, dynamic changes in frailty, and the quality of social engagement to inform targeted prevention and intervention strategies for vulnerable older adult populations. Clinically, the findings suggest that community health workers and social services should prioritize screening for psychological abuse and neglect—rather than physical or financial forms alone—when assessing anxiety risk among older adults, particularly in frailty screening contexts. Given the mediating role of physical frailty, interventions targeting physical function maintenance (e.g., structured exercise programs, fall prevention) may help reduce anxiety vulnerability among abuse-exposed older adults. The moderating role of social frailty highlights the importance of social integration and support networks; community-based programs that reduce social isolation (e.g., peer support groups, home visiting services) may buffer the emotional impact of abuse among socially frail elders. From a public health perspective, policies addressing elder abuse should move beyond incident reporting to incorporate holistic approaches that simultaneously target abuse prevention, frailty mitigation, and anxiety reduction in community-dwelling older adult.

## Data Availability

The raw data supporting the conclusions of this article will be made available by the authors, without undue reservation.
