# Supplementary material for: Elderly abuse and anxiety symptoms among older adults in China: the mechanism of physical frailty and social frailty
Source: Front Psychiatry. 2026 Oct 8;17:1813570. doi: 10.3389/fpsyt.2026.1813570 (PMC13687271; doi:10.3389/fpsyt.2026.1813570)
Supplement: Supplementary file 1 [file Table1.docx]

# **Supplementary Material**

This supplementary material contains the results of the Pearson correlation analysis (Supplementary Table 1), the propensity score matching (Supplementary Table 2), the subgroup analyses (Supplementary Tables 3-4), and the moderating effect analyses (Supplementary Tables 5-6).

## **Supplementary Table 1**

*Results of the Pearson Correlation Analysis.*

| **Variables** | **1** | **2** | **3** | **4** | **5** | **6** | **7** | **8** |
| --- | --- | --- | --- | --- | --- | --- | --- | --- |
| 1. Social frailty | 1 |  |  |  |  |  |  |  |
| 2. Physical frailty | -0.197** | 1 |  |  |  |  |  |  |
| 3. Anxiety | -0.106** | 0.689** | 1 |  |  |  |  |  |
| 4. Elderly abuse | -0.113** | 0.285** | 0.282** | 1 |  |  |  |  |
| 5. Psychological abuse | -0.102** | 0.270** | 0.255** | - | 1 |  |  |  |
| 6. Financial exploitation | -0.088** | 0.187** | 0.165** | - | - | 1 |  |  |
| 7. Neglect | -0.093** | 0.219** | 0.238** | - | - | - | 1 |  |
| 8. Physical abuse | -0.073** | 0.163** | 0.142** | - | - | - | - | 1 |

*Note: **P<0.01.*

## **Supplementary Table 2**

*Propensity score matching results.*

| **Methods** | **ATT** | **SE** | **t** | **P** | **95% CI** |
| --- | --- | --- | --- | --- | --- |
| Nearest neighbor Matching: 1:1 match | 1.06* | 0.432 | 2.45 | 0.015 | (0.21, 1.91) |
| Nearest neighbor Matching: 1:4 match | 1.02** | 0.386 | 2.65 | 0.008 | (0.26, 1.78) |
| Radius matching | 1.06* | 0.432 | 2.45 | 0.015 | (0.21, 1.91) |

*Note: *P<0.05, **P<0.01. ATT, Average Treatment Effect on the Treated; SE, standard error.*

## **Supplementary Table 3**

*Subgroup analysis among different sex.*

| **Variables (Males)** | **β** | **t** | **Variables (Females)** | **β** | **t** |
| --- | --- | --- | --- | --- | --- |
| Marital status | 0.727 | 2.816* | Urban-rural distribution | 1.358 | 4.298** |
| Chronic diseases | -0.954 | -2.233* | Elderly abuse | 2.379 | 2.289* |
| Psychological abuse | 1.653 | 3.279** | - | - | - |
| R | 0.432 |  | R | 0.405 |  |
| Adjusted R² | 0.173 |  | Adjusted R² | 0.154 |  |
| F | 14.427** |  | F | 15.510** |  |

*Note: *P<0.05, **P<0.001. This table only showed significant variables.*

## **Supplementary Table 4**

*Subgroup analysis among different urban-rural distribution.*

| **Variables (Urban)** | **β** | **t** | **Variables (Rural)** | **β** | **t** |
| --- | --- | --- | --- | --- | --- |
| Marital status | 0.788 | 2.852* | Chronic diseases | -1.144 | -2.988* |
| Psychological abuse | 1.171 | 2.647* | Elderly abuse | 3.418 | 2.667* |
| R | 0.318 |  | R | 0.421 |  |
| Adjusted R² | 0.091 |  | Adjusted R² | 0.166 |  |
| F | 9.698** |  | F | 15.599** |  |

*Note: *P<0.05, **P<0.001. This table only showed significant variables.*

## **Supplementary Table 5**

*The moderating effect between psychological abuse and anxiety symptoms.*

| **Variables** | **Model 1 β** | **Model 1 t** | **Model 2 β** | **Model 2 t** | **Model 3 β** | **Model 3 t** | **Model 4 β** | **Model 4 t** |
| --- | --- | --- | --- | --- | --- | --- | --- | --- |
| Sex | -0.088 | -0.504 | -0.088 | -0.508 | 0.324 | 1.454 | 0.355 | 1.609 |
| Age | -0.022 | -1.693 | -0.020 | -1.523 | 0.021 | 1.249 | 0.024 | 1.425 |
| Urban-rural distribution | 0.743 | 3.803** | 0.729 | 3.743** | 1.700 | 6.861** | 1.694 | 6.911** |
| Income per month | 0.232 | 2.119* | 0.226 | 2.063* | 0.069 | 0.485 | 0.102 | 0.728 |
| Education level | 0.085 | 1.383 | 0.069 | 1.131 | 0.067 | 0.845 | 0.066 | 0.848 |
| Living alone | 0.119 | 1.785 | 0.122 | 1.843 | 0.236 | 2.752* | 0.241 | 2.844* |
| Marital status | 0.254 | 1.753 | 0.250 | 1.723 | 0.427 | 2.283* | 0.417 | 2.257* |
| Chronic diseases | 0.149 | 0.613 | 0.097 | 0.401 | -0.732 | -2.367* | -0.792 | -2.588* |
| Psychological abuse | 0.397 | 3.197* | -0.931 | -1.838 | 1.178 | 7.558** | -1.939 | -3.349* |
| Physical frailty | 0.386 | 29.742** | 0.369 | 25.696** |  |  |  |  |
| Psychological abuse × Physical frailty |  |  | 0.042 | 2.704* |  |  |  |  |
| Social frailty |  |  |  |  | -0.064 | -2.922* | -0.115 | -4.909** |
| Psychological abuse × Social frailty |  |  |  |  |  |  | 0.153 | 5.585** |
| R² | 0.496 |  | 0.499 |  | 0.170 |  | 0.189 |  |
| F | 94.568** |  | 89.165** |  | 19.667** |  | 20.847** |  |

*Note: *P<0.05, **P<0.001.*

## **Supplementary Table 6**

*The moderating effect between neglect and anxiety symptoms.*

| **Variables** | **Model 1 β** | **Model 1 t** | **Model 2 β** | **Model 2 t** | **Model 3 β** | **Model 3 t** | **Model 4 β** | **Model 4 t** |
| --- | --- | --- | --- | --- | --- | --- | --- | --- |
| Sex | -0.082 | -0.471 | -0.089 | -0.511 | 0.349 | 1.564 | 0.351 | 1.582 |
| Age | -0.021 | -1.647 | -0.020 | -1.552 | 0.023 | 1.352 | 0.027 | 1.615 |
| Urban-rural distribution | 0.715 | 3.663** | 0.723 | 3.711** | 1.655 | 6.649** | 1.640 | 6.637** |
| Income per month | 0.217 | 1.981* | 0.202 | 1.850 | 0.016 | 0.111 | 0.059 | 0.416 |
| Education level | 0.085 | 1.387 | 0.072 | 1.185 | 0.072 | 0.913 | 0.052 | 0.664 |
| Living alone | 0.118 | 1.780 | 0.118 | 1.786 | 0.221 | 2.568* | 0.231 | 2.705* |
| Marital status | 0.284 | 1.961 | 0.283 | 1.961 | 0.516 | 2.760* | 0.552 | 2.971* |
| Chronic diseases | 0.160 | 0.662 | 0.133 | 0.549 | -0.715 | -2.305* | -0.752 | -2.442* |
| Neglect | 0.982 | 3.877** | -1.600 | -1.594 | 2.287 | 7.089** | -3.180 | -2.593* |
| Physical frailty | 0.386 | 30.043** | 0.371 | 26.245** |  |  |  |  |
| Neglect × Physical frailty |  |  | 0.083 | 2.659* |  |  |  |  |
| Social frailty |  |  |  |  | -0.062 | -2.821* | -0.100 | -4.302** |
| Neglect × Social frailty |  |  |  |  |  |  | 0.269 | 4.619** |
| R² | 0.498 |  | 0.500 |  | 0.166 |  | 0.179 |  |
| F | 95.243** |  | 89.766** |  | 19.100** |  | 19.518** |  |

*Note: *P<0.05, **P<0.001.*
